# Supplementary figures and images for: Assessing Heat Resistance and Selecting Heat-Resistant Individuals of Largemouth Bass (Micropterus salmoides) with Tiered Thermal Exposure
Source: Animals (Basel). 2025 Jan 8;15(2):128. doi: 10.3390/ani15020128 (PMC11758336; doi:10.3390/ani15020128)

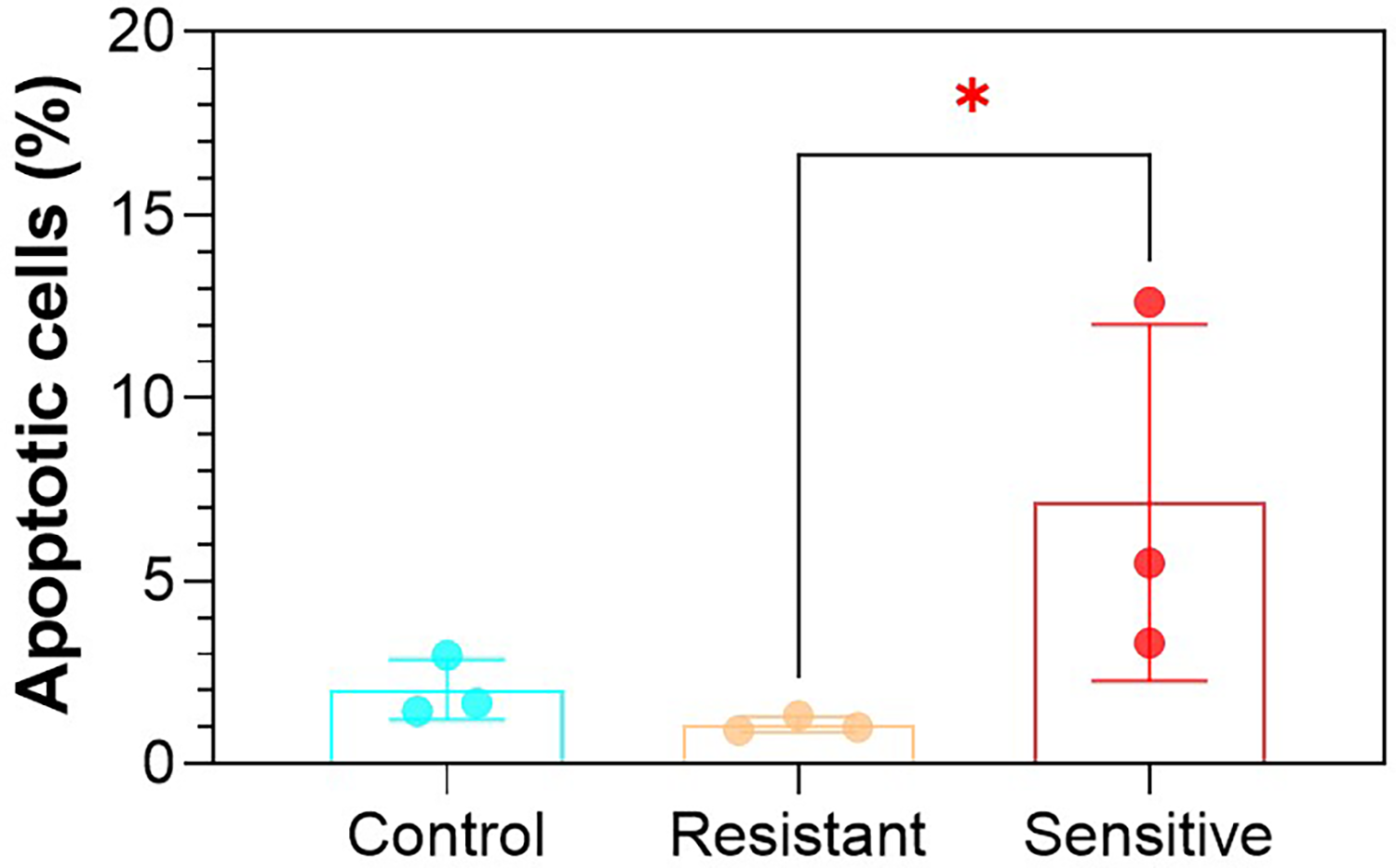

Supplement: Supplementary file 1 [file animals-15-00128-s001.zip › Supplementary materials/Figure S4.tif]
